# Evaluation of Acute and Convalescent Antibody Concentration against Pneumococcal Capsular Polysaccharides for the Diagnosis of Pneumococcal Infection in Children with Community-Acquired Pneumonia

**DOI:** 10.1097/INF.0000000000004185

**Published:** 2024-02

**Authors:** Michael J. Carter, Sonu Shrestha, Peter O’Reilly, Pallavi Gurung, Meeru Gurung, Stephen Thorson, Rama Kandasamy, Merryn Voysey, Elizabeth O’Mahony, Sarah Kelly, Imran Ansari, Ganesh Shah, Puja Amatya, Irina Tcherniaeva, Guy Berbers, David R. Murdoch, Andrew J. Pollard, Shrijana Shrestha, Dominic F. Kelly

**Affiliations:** 1 Department of Women and Children's Health, School of Life Course and Population Sciences, King's College London, London, UK; 2 Paediatric Intensive Care, St Mary's Hospital, Imperial College Healthcare NHS Trust, London, UK; 3 Oxford Vaccine Group, Department of Paediatrics, University of Oxford, Oxford, UK and NIHR Oxford Biomedical Research Centre, Oxford, UK; 4 Pediatric Research Group, Patan Academy of Health Sciences, Patan, Kathmandu, Nepal; 5 Discipline of Paediatrics and Child Health, School of Clinical Medicine, University of New South Wales, Sydney, Australia; 6 National Centre for Immunisation Research and Surveillance of Vaccine Preventable Diseases, Sydney, Australia; 7 National Institute for Public Health and the Environment (RIVM), Bilthoven, Netherlands; 8 Department of Pathology and Biomedical Science, University of Otago Christchurch, New Zealand

**Keywords:** Pneumococcal, Serology, Pneumonia, Children, Diagnostics

## Introduction

Modelling of data from randomized controlled trials has suggested that approximately one-third of children with pneumonia and radiographic consolidation have pneumococcal infection in settings without routine infant pneumococcal conjugate vaccination (PCV)^1^. However, microbiological techniques have limited accuracy to diagnose pneumococcal pneumonia for individual patients due to the inaccessibility of lung for sampling, pre-treatment with antibiotics, and prevalent nasopharyngeal (NP) carriage of pneumococci in healthy children^2^. Paired acute and convalescent serology to pneumococcal capsular polysaccharides (PS)^3^ or proteins^4^ from children with pneumococcal pneumonia may have diagnostic utility for pneumococcal pneumonia, but previous studies either do not use controls from the same disease population, and/or use arbitrary thresholds for defining a positive result. We evaluated the accuracy of serology to pneumococcal PS for the diagnosis of pneumococcal infection in children hospitalized with pneumonia in Kathmandu, Nepal during 2015-2017. 10-valent PCV (*Synflorix*, GSK) was introduced in the Nepal infant immunisation schedule in 2015, with no catch-up campaign. In children hospitalized with pneumonia in this setting, 73% and 78% of invasive pneumococcal disease (IPD) isolates were of serotypes covered by 10-valent PCV during 2005-2013 prior to 10-valent PCV introduction^5^, and NP carriage of any pneumococci was 36%, and of pneumococcal serotypes within 10-valent PCV was 14% during 2014-2015^6^.

## Methods

We sequentially recruited children 2 months to 14 years of age admitted to Patan Hospital, Kathmandu with a clinical diagnosis of pneumonia. All children had chest radiographs, full blood count and C-reactive protein (CRP) measurement, culture of blood (Bactec PedsPlus culture bottles, BD, USA; aerobic culture in 5% CO_2_ at 35–37°C) and NP sampling with flocked swabs (ThermoFisher Scientific, UK) for pneumococcal culture and polymerase chain reaction (PCR) detection of respiratory viruses (NxTAG Luminex Respiratory Pathogen Panel, Luminex Corp, USA) within 48 hours of admission. Serotyping of pneumococci used the Quellung method (Statens Serum Institut, Denmark)^6^. Convalescent sampling for serum of recruited children was done 6–8 weeks following admission.

Samples were included for serological testing if paired acute and convalescent samples were available. We defined a series of comparator groups by *a priori* probability of having true pneumococcal pneumonia. Of note, NP carriage of serotype 1 or 5 pneumococci (but not other serotypes) has a high positive predictive value for IPD in this setting^5^. Participants were grouped as follows, definite pneumococcal pneumonia (pneumococci cultured from blood or pleural fluid), probable pneumococcal pneumonia (CRP concentration ≥60 mg/l and NP carriage of serotype 1 or 5), probable bacterial pneumonia (CRP concentration ≥60 mg/l and *no* NP carriage of serotype 1 or 5), unknown pneumonia etiology, RSV pneumonia only (CRP concentration <60 mg/l and NP carriage of RSV), and definite other bacterial pneumonia (other bacterial pathogen cultured from blood). All participant-samples with definite and probable pneumococcal pneumonia, RSV pneumonia and definite other bacterial pneumonia, and a randomized selection of probable bacterial pneumonia, and unknown pneumonia were included. Serum concentration of IgG to pneumococcal PS contained in the 13-valent PCV was measured using a fluorescence-based multiplex immunoassay (FMIA, RIVM, Netherlands)^7^. We investigated whether change in absolute PS-specific IgG concentration (delta concentration) or fold change in PS-specific IgG concentration between acute and convalescent samples, or maximum PS-specific IgG convalescent concentration, was associated with pneumococcal pneumonia. As a sensitivity analysis we also evaluated serotype 1 and serotype 5 specific values in children with pneumococcal pneumonia caused by serotypes 1 and 5, with children with RSV pneumonia only as a comparator group.

## Results

Between 2015 and 2017, 897 children were sequentially recruited to the overall study. Of all children recruited, median age was 1.5 years (interquartile range, IQR, 0.7–3.1, years) and 528 (59%) children were male. Of these children, 454 (51%) returned for convalescent sampling (median 47, IQR, 37–62, days after acute sampling), and 221 (49%) children with paired serum samples entered further analysis. Of these 221 children, median age was 1.7 (IQR 0.7–3.7) years, 133 (60%) children were male and 58 (26%) had received ≥2 doses of 10-valent PCV according to caregiver information (Supplementary Table 1). On admission chest radiographs, 80 (36%) children had alveolar consolidation or effusion. Median CRP concentration at admission was 58 (IQR 6.8–109) mg/L. Eight children were classified as definite pneumococcal pneumonia (median age 5.0, IQR 3.8–6.6, years), 11 children as probable pneumococcal pneumonia (median age 4.7, IQR 3.3–7.7, years), 90 children as probable bacterial (median age 2.7, IQR 1.4–5.5, years), 68 children as RSV pneumonia (median age 0.7, IQR 0.4–1.5, years), 5 children as other bacterial pneumonia (median age 0.7, IQR 0.7–0.9, years) and 39 children as unknown (median age 1.3, IQR 0.8–2.9, years). Of children with definite pneumococcal pneumonia, 5 children had pneumococci isolated from blood (2 each of serotypes 1 and 5, and 1 serotype 6C) and 3 children had pneumococci isolated from pleural fluid (1 serotype 19A, 1 serotype 6B, 1 not serotyped).

**Figure 1:**
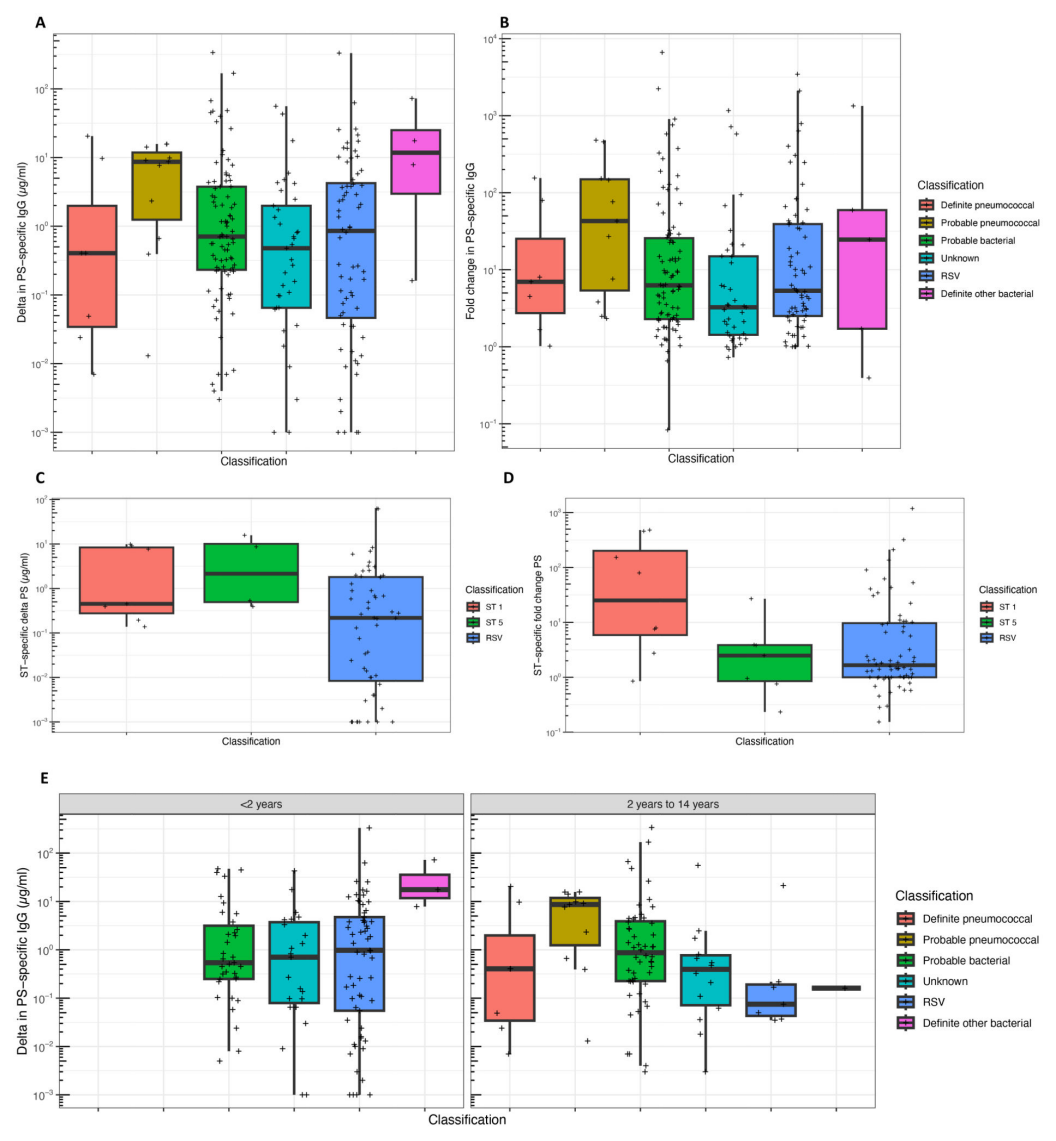
Serum IgG to pneumococcal polysaccharides in children with acute pneumonia. In all plots, the y axis is on a log_10_ scale and points represent the greatest of change in concentration from acute to convalescent samples (expressed as “delta“), or greatest fold change for and of the 13 polysaccharides assayed in an individual child. A: Maximum change in concentration between acute and convalescent samples for each diagnostic group. B: Maximum fold change in concentration between acute and convalescent samples for each diagnostic group. C: Maximum change in concentration between acute and convalescent samples for children with pneumonia associated with serotype 1 pneumococci, with pneumonia associated with serotype 5 pneumococci, or RSV pneumonia (as a comparator). D: Maximum fold change in concentration between acute and convalescent samples for children with pneumonia associated with serotype 1 pneumococci, with pneumonia associated with serotype 5 pneumococci, or RSV pneumonia (as a comparator). E: Maximum change in concentration between acute and convalescent samples for each diagnostic group stratified by age group. All statistical comparisons were made from the raw (non-logged) data.

There were no significant differences in the acute to convalescent change in concentration (delta concentration) of IgG to pneumococcal PS by classification of pneumonia etiology (Kruskal-Wallis Test, p=0.44, Figure 1A; multiple pairwise comparisons with Wilcoxon Test and Benjamin-Hochberg adjustment for multiple comparisons, p>0.15 for all comparisons), and there were no significant differences in acute to convalescent fold change of IgG to pneumococcal PS by classification of pneumonia etiology (p=0.45, Figure 1B; p>0.10 for pairwise comparisons). When analysis was limited to patients with serotype 1 (8 children) and serotype 5 (7 children) definite or probable pneumococcal pneumonia there were no differences in delta concentration to the relevant PS in comparison with children with RSV pneumonia (p=0.27 and p=0.36, respectively, Figure 1C). In children with serotype 5 definite or probable pneumococcal pneumonia there was no difference in fold change of IgG to pneumococcal PS5 (p=0.79), but children with serotype 1 definite or probable pneumococcal pneumonia had significantly higher fold change of IgG to pneumococcal PS1, in comparison with children with RSV pneumonia (p=0.01, Figure 1D). No children <2 years of age had definite or probable pneumococcal pneumonia. In children aged ≥2 years there were no significant differences in delta concentration of IgG to pneumococcal PS by classification of pneumonia etiology (Figure 1E, p>0.15 for pairwise comparisons). Similar results were obtained by maximum convalescent IgG concentration (Supplementary Figure 1).

## Discussion

Analyses of data from Belgium^3^, Finland^4^, and Brazil^4^ have suggested that paired serological testing may accurately diagnose pneumococcal pneumonia in children. Tuerlinckx *et al.^3^* quantified PS-specific IgG and IgA concentrations in acute and convalescent serum from Belgian children with pneumonia. Among children with culture-proven pneumococcal pneumonia, 83% of paired samples met the pre-defined "positive" threshold of ≥3-fold increase in PS-specific IgG concentration. Among children with non-proven pneumococcal pneumonia, 55% of paired samples met this threshold; no other control samples were analyzed. As with data from Nepal, PS-specific IgG concentration in these Belgian data was associated with increasing age, with only 50% of definite pneumococcal pneumonia cases and 13% of suspected/possible pneumococcal cases <2 years of age meeting the positive threshold. Different age distributions between the Nepal (median 1.7 years) and Belgian studies (median 4.0 years), or different distributions of first colonisation with pneumococcal serotype, may have contributed to differences in apparent prevalence of "positive" pneumococcal serology.

Given the diversity of pneumococcal PS, assay of IgG to pneumococcal proteins may improve the sensitivity of serological testing. Andrade *et al.*^4^ evaluated the use of paired serology to 8 pneumococcal proteins to discriminate between pneumococcal pneumonia in Brazil (13 children, median age 14 months, non-PCV vaccinated) and a viral pharyngitis control group in Finland (23 children, median age 37 months, PCV-vaccinated). Receiver-operator characteristic curves yielded areas-under-the-curve (AUC) of 0.67-0.93. However, the use of controls from a different population and disease, may have confounded these results. We previously extended this work by examining the production of IgG to five conserved pneumococcal proteins in the antibody-in-lymphocyte-supernatant (ALS) assay in Nepali children^8^, finding that lymphocyte production of IgG to pneumococcal proteins discriminated between pneumococcal and non-pneumococcal pneumonia with AUC of 0.60–0.85. However, when stratified into children ≥2 years of age, there were no significant differences in protein-specific IgG production between pneumococcal and non-pneumococcal pneumonia. As with PS-specific IgG concentration, production of protein-specific IgG was associated with increasing age.

In the absence of a Gold-standard, we used comparator groups from the same population of children with other pneumonia etiologies to assess a diagnostic test. We have previously shown that NP carriage of serotype 1 or 5 pneumococci may enrich this cohort for pneumococcal pneumonia^6^. Despite this, the small number of children with definite or probable pneumococcal pneumonia limited our study power, particularly in children <2 years of age. In addition, we sampled convalescent serum at a median 47 days, while other studies sampled convalescent serum at 3–4 weeks^3^ or 2–5 weeks^4^, following admission. Our data may therefore represent antibody concentrations that are already declining. Measurement of IgG to specific pneumococcal PS, has been used to assess population immunity^9^ and combined with functional antibody studies to assess correlates of PCV-mediated protection^10^. However, in this cohort, it was not useful to diagnose pneumococcal pneumonia for individual patients.

## Supplementary Material

Supplementary Information
