# Supplementary material for: Evaluation of Acute and Convalescent Antibody Concentration Against Pneumococcal Capsular Polysaccharides for the Diagnosis of Pneumococcal Infection in Children with Community-Acquired Pneumonia
Source: Pediatr Infect Dis J. 2024 Jan 11;43(2):e67–70. doi: 10.1097/INF.0000000000004185 (PMC10789377; doi:10.1097/INF.0000000000004185)
Supplement: Supplementary file 1 [file inf-43-e67-s001.pdf]

SUPPLEMENTARY INFORMATION

Supplementary Table 1.

	Pneumococcal pneumonia		**Probable bacterial pneumonia**	**Unknown**	**RSV pneumonia**	**Definite other bacterial pneumonia**
	**Definite pneumococcal pneumonia**	**Probable pneumococcal pneumonia**				
n	8	11	90	39	68	5
Age (years; median, IQR)	5.0 (3.8–6.6)	4.7 (1.4–5.5)	2.6 (1.4–5.5)	2.1 (0.8–2.9)	0.7 (0.4–1.5)	0.7 (0.7–0.9)
<2 years of age	0	0	37 (41%)	25 (64%)	61 (90%)	4 (80%)
2–5 years of age	4 (50%)	6 (55%)	28 (31%)	11 (28%)	7 (10%)	0
≥5–14 years of age	4 (50%)	5 (46%)	25 (28%)	3 (7%)	0	1 (20%)
Female sex	1 (13%)	4 (36%)	40 (44%)	14 (36%)	26 (38%)	3 (60%)
Length of illness (days; median, IQR)	4 (3–5)	4 (3–6)	4 (2–7)	4 (3–7)	4 (3–5)	4 (2–4)
Acute to convalescent sampling period (days; median, IQR)	45 (34–58)	36 (35–44)	47 (37–64)	47 (36–60)	48 (42–59)	49 (38–60)
Primary endpoint pneumonia	8 (100%)	10 (91%)	54 (60%)	17 (44%)	0	1 (20%)
NP pneumococcal carriage	4 (50%)	11 (100%)	31 (34%)	10 (26%)	24 (35%)	2 (40%)
NP 13-valent PCV serotype carriage	4 (50%)	11 (100%)	14 (16%)	3 (8%)	10 (15%)	0
NP serotype 1 or 5 pneumococcal carriage	2 (25%)	11 (100%)	0	0	0	0
CRP concentration (mg/l; median, IQR)	216 (151–238)	185 (151–238)	103 (84–176)	15 (2–26)	4 (1–16)	15 (13–88)
NP RSV carriage	0	0	5 (6%)	3 (9%)	68 (100%)	2 (50%)

Supplementary Figure 1. Serum IgG to pneumococcal polysaccharides in children with acute pneumonia.

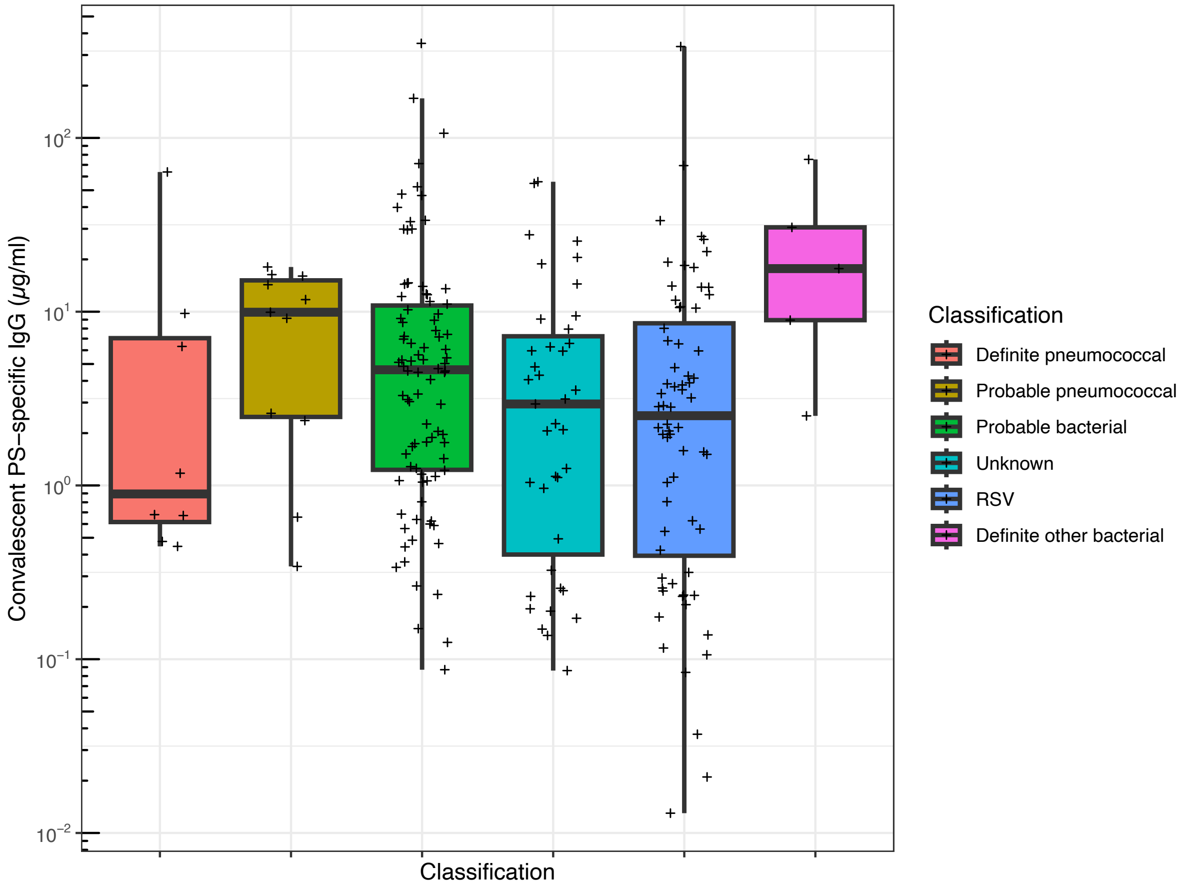

REFERENCES

1 Cherian, T. *et al.* Standardized interpretation of paediatric chest radiographs for the diagnosis of pneumonia in epidemiological studies. *Bull WHO* **83**, 353-359 (2005).
